# Assessment of Heart Failure Patients’ Interest in Mobile Health Apps for Self-Care: Survey Study

**DOI:** 10.2196/14332

**Published:** 2019-10-29

**Authors:** Albert Sohn, William Speier, Esther Lan, Kymberly Aoki, Gregg Fonarow, Michael Ong, Corey Arnold

**Affiliations:** 1 Department of Radiological Sciences University of California, Los Angeles Los Angeles, CA United States; 2 Department of Cardiology University of California, Los Angeles Los Angeles, CA United States; 3 Pathology & Laboratory Medicine University of California, Los Angeles Los Angeles, CA United States

**Keywords:** mHealth, patient-reported outcome, heart failure, self-care, patient monitoring

## Abstract

**Background:**

Heart failure is a serious public health concern that afflicts millions of individuals in the United States. Development of behaviors that promote heart failure self-care may be imperative to reduce complications and avoid hospital re-admissions. Mobile health solutions, such as activity trackers and smartphone apps, could potentially help to promote self-care through remote tracking and issuing reminders.

**Objective:**

The objective of this study was to ascertain heart failure patients’ interest in a smartphone app to assist them in managing their treatment and symptoms and to determine factors that influence their interest in such an app.

**Methods:**

In the clinic waiting room on the day of their outpatient clinic appointments, 50 heart failure patients participated in a self-administered survey. The survey comprised 139 questions from previously published, institutional review board–approved questionnaires. The survey measured patients’ interest in and experience using technology as well as their function, heart failure symptoms, and heart failure self-care behaviors. The Minnesota Living with Heart Failure Questionnaire (MLHFQ) was among the 11 questionnaires and was used to measure the heart failure patients’ health-related quality of life through patient-reported outcomes.

**Results:**

Participants were aged 64.5 years on average, 32% (16/50) of the participants were women, and 91% (41/45) of the participants were determined to be New York Heart Association Class II or higher. More than 60% (30/50) of the survey participants expressed interest in several potential features of a smartphone app designed for heart failure patients. Participant age correlated negatively with interest in tracking, tips, and reminders in multivariate regression analysis (*P*<.05). In contrast, MLHFQ scores (worse health status) produced positive correlations with these interests (*P*<.05).

**Conclusions:**

The majority of heart failure patients showed interest in activity tracking, heart failure symptom management tips, and reminder features of a smartphone app. Desirable features and an understanding of factors that influence patient interest in a smartphone app for heart failure self-care may allow researchers to address common concerns and to develop apps that demonstrate the potential benefits of mobile technology.

## Introduction

### Background

Heart failure is a complex clinical syndrome characterized by the impairment of the heart’s function to fill or eject blood [1,2]. It is a major global health problem with an estimated prevalence of 6.5 million adults in the United States [3] and 37.7 million people worldwide [4]. Every year in the United States, there are approximately 1 million new cases of heart failure and 330,000 heart failure—related deaths [3]. Projections suggest that heart failure’s prevalence will increase by 46% between 2012 and 2030 [5]. Its total cost, which includes the expense of health care services, medications, and sick leave, may reach US $69.7 billion by 2030, a 127% increase from roughly US $30.7 billion in 2012 [5].

Several cohort studies have indicated that the prevalence of heart failure increases significantly with age. In the Framingham Study by Ho et al [6], the prevalence was 0.8% in both men and women aged between 50 and 59 years before rising to 6.6% in men and 7.9% in women aged between 80 and 89 years. Similarly, the Rotterdam Study by Mosterd et al [7] showed a prevalence of 1% in the age group of 55 to 64 years, whereas it surpassed 10% in individuals aged 85 years or older. Much like its prevalence, incidence of heart failure is substantially higher in the elderly. In contrast to the annual incidence rates of 0.3% in men and 0.2% in women aged between 50 and 59 years, rates were 2.7% and 2.2%, respectively, in those aged between 80 and 89 years [6]. The cardiovascular health study by Huffman et al [8] that focused on individuals aged 65 years or older approximated an incidence of 19.3 per 1000 person-years.

Owing to the increasing prevalence of heart failure and rising financial implications, forming efficient heart failure prevention and treatment strategies is imperative. Currently, clinicians counsel heart failure patients on evidence-based recommendations outlined in clinical practice guidelines, which include taking prescription drugs, exercising, monitoring daily weight, and restricting sodium intake [9]. However, divergence from these guidelines contributes to hospital re-admission rates that surpass 20% within the first 30 days of discharge [10,11] and approach 50% within 6 months of discharge [12], with a substantial proportion of the 30-day rehospitalizations considered preventable [13].

### Objectives

As heart failure patients show poor adherence to self-care behaviors, mobile health (mHealth) has emerged as a potential solution to improve their health outcomes and quality of care. mHealth is defined as the application of mobile technology [14,15], including software apps on mobile devices [16] and wireless sensors such as activity trackers [17]. These technological developments monitor activity and provide reminders of self-care behaviors and heart failure symptoms, which may be difficult for patients to ascertain [16]. Moreover, activity trackers are minimally invasive options that may also be preferable because of individuals’ relatively high adherence to wearing them upon recommendation. In a previous study performed by members of our team, adherence rates for wearing activity trackers were observed to be as high as 90% [18]. The purpose of this study was to assess patient interest, specifically needs and preferences, regarding their heart failure self-care and their perceptions regarding a smartphone app integrated with home monitoring sensors. Results were analyzed to achieve the secondary end point of this study, which was to determine the factors that influence their interest.

## Methods

### Recruitment

From February 2018 to September 2018, study personnel collaborated with internal medicine, cardiomyopathy, and cardiology outpatient clinics to prescreen all patients diagnosed with heart failure at a university-based health system. Heart failure patients aged between 50 and 80 years were eligible to participate in this anonymous study if they were scheduled for an appointment at any of the 3 outpatient clinics. Exclusion criteria included having a cognitive (eg, dementia) disability, being unable to communicate in English, and having visual or auditory impairments to the extent that a smartphone could not be used.

Research personnel contacted potential participants over the phone, provided additional information about the study, and conducted the verbal consent process with those who were interested in participating. In the clinic waiting room, an informational sheet that described the study was given to those who consented to participate. The research team asked the participants to complete the survey before their scheduled appointment and informed them that omitting answers to any questions was permitted. Enrolled subjects received a US $20 gift card.

Upon enrolling in the study, each participant’s New York Heart Failure Association (NYHA) classification and ejection fraction (EF) was noted. The NYHA classification categorizes heart failure patients by considering their symptoms during physical activity [19]. EF is a measurement that reports the heart’s degree of function by monitoring the percentage of blood leaving the left ventricle when it contracts. These data were recorded to describe the patients’ heart failure according to the severity of their symptoms and limitations.

### Survey Questions

The survey comprised 15 sections, all written in American English. A total of 4 sections comprised questions relating to sociodemographic information, interest in specific smartphone app features, preferences regarding specific smartphone app notifications, and experience using technology. The section pertaining to interest in specific smartphone app features for heart failure self-care management evaluated the participants’ interests using a 5-point Likert scale [20]. It included questions regarding symptom tracking, tips, and reminders (Multimedia Appendix 1). Each participant’s responses to questions in these groups were averaged for data analysis. The section concerning notification preferences instructed subjects to indicate how often they would like to receive reminders and information related to heart failure self-care: never, once a day, every 12 hours, every 6 hours, every 4 hours, or every 2 hours (Multimedia Appendix 1). To determine the participants’ experience with technology, 12 *yes or no* questions from the Health Information National Trends Survey were asked (Multimedia Appendix 1) [21].

The remaining sections included questions regarding function, heart failure symptoms, and heart failure self-care behaviors. The participants’ function and behaviors were detailed using the following institutional review board–approved questionnaires: Minnesota Living with Heart Failure Questionnaire (MLHFQ), Self-Care of Heart Failure Index (SCHFI), shortened version of the Seattle Angina Questionnaire (SAQ-7), shortened version of the Kansas City Cardiomyopathy Questionnaire (KCCQ-12), Patient-Reported Outcomes Measurement Information System (PROMIS) Global Health, and PROMIS Physical Function short form (SF). Symptoms were measured using a variety of PROMIS questionnaires: Fatigue SF, Anxiety SF, Depression SF, Sleep Disturbance SF, and Social Isolation SF. Scores from these questionnaires represented patient-reported outcomes (PROs), which are reports of a patient’s health status directly from the patient. PROs were used to describe the study population because patients were recruited irrespective of the time of their heart failure diagnoses. Along with participants’ demographics, MLHFQ scores were of particular interest as they represented heart failure patients’ health-related quality of life (HRQOL), which is a factor that might influence their interests in features of a smartphone app.

### Scoring

The 21-item MLHFQ is among the most widely used patient-oriented measurements of HRQOL [22]. It accounted for 3 ways heart failure affected the participants: physical, emotional, and socioeconomic. Although there is no scale for the socioeconomic score, physical (0-40) and emotional (0-25) scores were calculated by summation of corresponding responses. Lower scores signified better HRQOL, whereas higher scores signified worse HRQOL in regard to physical and emotional well-being [22]. A total score was also generated by addition of all 21 responses, resulting in a possible range of 0 to 105. Scores were classified as good (<24), moderate (24-45), and poor (>45) HRQOL [22].

SCHFI is a 22-item questionnaire that assesses the patient’s ability to care for their heart failure via 3 subscales: maintenance, management, and confidence [23]. For each subscale, the raw score was calculated by summation of corresponding responses. Raw scores were then standardized to a 0 to 100 range, with higher scores indicating better self-care. Management scores were calculated only if heart failure patients acknowledged having trouble breathing or ankle swelling within the past month of taking this survey. For all sections of the SCHFI, scores ≥70 proposed adequate self-care [23].

The SAQ-7 and KCCQ-12 also assessed the HRQOL of patients with respect to angina and heart failure, respectively [24,25]. Scores for both questionnaires were calculated by summation of all 7 and 12 responses, respectively, and by standardization of those values to a 0 to 100 range. Scores were classified as poor (0-24), fair (25-49), good (50-74), and excellent (75-100) HRQOL [24,25].

PROMIS questionnaires are publicly available individual-centered measures of PROs [26,27]. The aforementioned physical and mental health questionnaires were administered to heart failure patients to assess their function and symptoms. Raw scores were computed by addition of all corresponding responses and conversion of those values to *t* scores, which were standardized scores set to a mean of 50 and a standard deviation of 10 [26,27]. Function scores ≥40 were normal, whereas scores <40 denoted moderate to severe adverse health effects. Symptom scores ≤60 were normal, whereas scores >60 represented moderate to severe adverse health effects [26,27].

### Statistical Analysis

Before calculating raw scores, questionnaires were examined for completion. For any missing items, the mean of the participant’s responses from the same questionnaire was substituted [28]. The cohort was characterized using proportions, means, SDs, medians, and interquartile ranges (IQRs). Summaries of responses and scores, if applicable, for each questionnaire were reported. Linear regression analyses, including multivariate regression analysis, were performed with the participants’ age and MLHFQ scores as the independent variables to quantify the linear relationships with their interest in smartphone app features. For all analyses, a significance level of .05, which corresponded to a 95% CI, was used to determine statistical significance.

## Results

### Demographics

Over the 7-month period, a total of 95 eligible heart failure patients were contacted. Of the 95 qualified patients, 50 consented to participate in this study (Table 1). However, 1 participant only completed the demographics section of the survey.

The participants’ mean age was 64.5 years (SD 8.3; range 50-78). Most participants were men (34/50, 68%), of non-Hispanic or non-Spanish origin (40/49, 82%), and white (32/48, 67%). Of the participants, 38% (19/50) had received a bachelor’s degree or higher, whereas for 18% (9/50), a high school degree was their highest level of education. As for annual household income, the proportions of individuals whose families earned less than US $50,000 (23/50, 46%) and more than US $50,000 (27/50 54%) were fairly similar. Although 91% (31/45) of the participants were determined to be NYHA Class II or higher, 62% (31/50) had EFs less than 50%. Neither NYHA class nor EF produced statistically significant associations with their interests in potential features of a smartphone app.

**Table 1:** Demographics of study population.

Characteristic		Value
Age (years; n=50), mean (SD)		64.5 (8.3)
**Sex (n=50), n (%)**		
	Male	34 (68)
	Female	16 (32)
**Hispanic or Spanish origin (n=49), n (%)**		
	No	40 (82)
	Yes	9 (18)
**Race or ethnicity (n=48), n (%)**		
	White	32 (67)
	Black or African American	11 (23)
	Asian	5 (10)
	American Indian or American Native	0 (0)
	Native Hawaiian or other Pacific Islander	0 (0)
**Education (n=50), n (%)**		
	High school	9 (18)
	Some college, associate degree, or trade school	22 (44)
	Bachelor’s degree	10 (20)
	Master’s degree or above	9 (18)
**Annual income (US $; n=50), n (%)**		
	0-25,000	15 (30)
	25,001-50,000	8 (16)
	50,001-75,000	8 (16)
	≥75,001	19 (38)
**New York Heart Association class (n=45), n (%)**		
	I	4 (9)
	II	26 (58)
	III	15 (33)
	IV	0 (0)
**Ejection fraction (n=50), n (%)**		
	≤40%	28 (56)
	41%-49%	3 (6)
	≥50%	19 (38)

### App Interest

More than 60% of the participants were somewhat interested or very interested in a smartphone app that provides information related to symptoms (identification 31/48, 65%, and tips 35/48, 73%), medication or treatment (side effects 33/48, 69%), activity (steps 33/48, 69%, and exercise 31/48, 65%), and sleep (patterns 32/46, 67%, and tips 31/47, 66%; Table 2). On the other hand, more than a quarter of the participants expressed little to no interest in documenting their mood (17/48, 35%) or receiving tips to improve their mood (14/48, 29%). Moreover, 30 participants answered somewhat interested or very interested for both symptom-related statements (Multimedia Appendix 2). Of those 30 participants, 28 (28/30, 93%) owned a smartphone and 10 (10/30, 33%) owned an activity tracker or a smartwatch. Of the 28 participants who expressed interest (somewhat interested or very interested) in both activity-related statements, 26 (26/28, 93%) owned a smartphone and 11 (11/28, 39%) owned an activity tracker or a smartwatch. There were 27 participants who showed interest in both items regarding sleep. Of these, 24 (24/27, 89%) owned a smartphone and 11 (11/27, 41%) owned an activity tracker or a smartwatch.

**Table 2:** Patients’ answers to the Heart Failure Self-Care Management Application Interest questionnaire.

Statement	n	No interest, n (%)	Not very interested, n (%)	Neutral, n (%)	Somewhat interested, n (%)	Very interested, n (%)
Symptom identification, such as noticing swelling in your ankles or legs	48	7 (15)	2 (4)	8 (17)	11 (23)	20 (42)
Providing symptom management tips	48	7 (15)	1 (2)	5 (10)	13 (27)	22 (46)
Providing medication reminders	48	9 (19)	4 (8)	9 (19)	9 (19)	17 (35)
Documenting when you experience side effects from medication or treatment	48	5 (10)	2 (4)	8 (17)	10 (21)	23 (48)
Documenting your level of activity or number of steps	48	6 (13)	4 (8)	5 (10)	9 (19)	24 (50)
Providing reminders to exercise	48	7 (15)	2 (4)	8 (17)	10 (21)	21 (44)
Documenting your sleep patterns	46	5 (11)	3 (7)	6 (13)	10 (22)	22 (48)
Providing tips to get better sleep	47	6 (13)	4 (9)	6 (13)	6 (13)	25 (53)
Documenting your mood	48	9 (19)	8 (17)	8 (17)	7 (15)	16 (33)
Providing tips to improve your mood	48	9 (19)	5 (10)	10 (21)	8 (17)	16 (33)

### Reminders

Between 80% and 90% of the participants indicated their desire to receive reminders at least once per day for all but medication reminders, which was 71% (34/48; Table 3). Once a day was the most popular response for the other 5 features. The proportion exceeded 50% for symptom management tips (27/49, 55%), activity or steps (25/49, 51%), exercise reminders (27/49, 55%), and sleep tips (29/49, 59%).

### Access to Technology

The majority of participants had access to technology. Only 24 (24/49, 49%) participants owned a tablet, and 44 (44/49, 90%) participants owned a smartphone (Table 4). In addition, high proportions of participants had access to the internet through a cellular network (41/49, 84%) or a wireless network (43/49, 88%). Most participants also had experience using their smartphone (42/44 smartphone owners, 96%) and accessing the internet or their email account(s) (44/49, 90%). Fewer patients had activity trackers and smartwatches as only 14/49 (29%) participants owned one and 9/14 (64%) participants used it regularly. Ownership of an activity tracker or smartwatch was not related to income, as half of them earned a household income that surpassed US $75,001 annually.

**Table 3:** Patients’ answers to Heart Failure Self-Care Management Application Engagement questionnaire.

Statement	n	Never, n (%)	Once a day, n (%)	Every 12 hours, n (%)	Every 6 hours, n (%)	Every 4 hours, n (%)	Every 2 hours, n (%)
Notify you of symptoms	49	9 (18)	20 (41)	8 (16)	3 (6)	4 (8)	5 (10)
Provide you with symptom management tips	49	6 (12)	27 (55)	9 (18)	1 (2)	3 (6)	3 (6)
Provide you with medication reminders	48	14 (29)	12 (25)	9 (19)	4 (8)	3 (6)	6 (12)
Provide you with your level of activity/number of steps	49	5 (10)	25 (51)	3 (6)	4 (8)	6 (12)	6 (12)
Provide you with exercise reminders	49	5 (10)	27 (55)	5 (10)	3 (6)	5 (10)	4 (8)
Provide you with sleep tips	49	8 (16)	29 (59)	6 (12)	2 (4)	0 (0)	4 (8)

**Table 4:** Patient answers to Health Information Nation Trends Survey.

Question	n	No, n (%)	Yes, n (%)
Do you ever access the internet or World Wide Web or send and receive email?	49	5 (10)	44 (90)
When you use the internet, do you ever access it through a regular dial-up telephone line?	49	48 (98)	1 (2)
When you use the internet, do you ever access it through broadband such as digital subscriber line, cable, or fiber optic service?	49	15 (31)	34 (69)
When you use the internet, do you ever access it through a cellular network (ie, phone and third- or fourth-generation cellular network technology)?	49	8 (16)	41 (84)
When you use the internet, do you ever access it through a wireless network (wireless fidelity)?	49	6 (12)	43 (88)
Do you own a tablet?	49	25 (51)	24 (49)
Do you own a smartphone?	49	5 (10)	44 (90)
If so, do you use your smartphone at least once daily?	43	1 (2)	42 (97)
Do you own a cell phone? (skip if yes answer to smartphone)	5	1 (20)	4 (80)
If so, are you comfortable using the cell phone?	4	1 (25)	3 (75)
Do you own an activity tracker/smartwatch?	49	35 (71)	14 (29)
If so, do you wear it daily?	14	5 (36)	9 (64)

### Patient-Reported Outcomes

The median MLHFQ score was 52 (IQR 24-75; Table 5), which corresponded to a poor HRQOL for the average participant. On the other hand, SAQ (median 68, IQR 55-84) and KCCQ (median 61, IQR 47-80) median scores suggested a good HRQOL in relation to angina and heart failure, respectively. The median SCHFI maintenance (median 70, IQR 60-81) and SCHFI confidence (median 72, IQR 50-83) scores revealed adequate ability to perform maintenance behaviors and adequate confidence level for the average participant. Of the 49 participants, 28 (57%) indicated recent breathing complication or ankle swelling (Table 5), which qualified them to complete the management section of the SCHFI questionnaire. Similar to the other section scores, the median SCHFI management score (median 70, IQR 50-85) indicated adequate ability to manage heart failure. Median scores for all PROMIS questionnaires were within the normal range, except for Physical Function SF (median 38, IQR 34-43), which denoted moderate adverse health implications.

In the Heart Failure Self-Care Management Application Interest questionnaire, 67% (32/48) said they were interested in tracking, whereas 65% (31/48) said they were interested in tips and 73% (35/48) said they were interested in reminders (Table 2). Age correlated significantly with interest in each of the 3 features of the smartphone app (*P*=.001, *P*=.002, and *P*=.001, respectively). In contrast to age, MLHFQ scores (Table 5) generated positive correlations with their interests. These correlations were also statistically significant (*P*=.003, *P*<.001, and *P*=.004, respectively). Similarly, when multivariate regression analyses were performed with age and MLHFQ scores, they generated negative coefficients for age and positive coefficients for MLHFQ scores. Moreover, both identifiers achieved statistically significant results with tracking (*P*=.007 and .02, respectively), tips (*P*=.01 and .002, respectively), and reminders (*P*=.007 and .02, respectively).

No relationship between age and frequency of the 6 different reminders (Table 3) was statistically significant: symptoms, symptom management tips, medication reminders, activity/steps, exercise reminders, and sleep tips (*P*=.09, *P*=.26, *P*=.09, *P*=.09, *P*=.13, and *P*=.40, respectively).

**Table 5:** Patient-reported outcomes.

Questionnaire		n	Median score (IQR)
**Minnesota Living with Heart Failure Questionnaire**			
	Physical score	49	19 (12-32)
	Emotional score	48	10 (2-20)
	Total score	49	52 (24-75)
**Self-Care of Heart Failure Index**			
	Maintenance	49	70 (60-81)
	Management	28	70 (50-85)
	Confidence	49	72 (50-83)
Seattle Angina Questionnaire		49	68 (55-84)
Kansas City Cardiomyopathy Questionnaire		49	61 (47-80)
**PROMIS^a^ Global Health**			
	Physical	49	42 (35-51)
	Mental	49	48 (44-51)
PROMIS Physical Function		49	38 (34-43)
PROMIS Fatigue		49	57 (46-63)
PROMIS Anxiety		49	54 (39-61)
PROMIS Depression		49	52 (41-61)
PROMIS Sleep Disturbance		49	52 (46-60)
PROMIS Social Isolation		49	40 (35-50)

^a^PROMIS: Patient-Reported Outcomes Measurement Information System.

## Discussion

### Principal Findings

The results indicate that 38 out of 48 survey participants (79%) were interested in at least one of the following features of a smartphone app to assist their heart failure management: symptoms, medication or treatment side effects, activity/steps, and sleep. Consequently, this study suggests the prospect of heart failure patients utilizing a smartphone app to self-monitor their condition while also receiving tips and reminders related to heart failure. Access to and experience with technology should not pose major concerns to its potential, as 43 out of 49 participants (88%) owned a smartphone and had access to the internet.

MLHFQ score and age were 2 factors that correlated the participants’ degree of interest. Their responses to questions in this survey and subsequent scores imply that many experienced adverse health outcomes because of their heart failure. The statistically significant positive correlations between their MLHFQ score and interest in tracking, tips, and reminders show that heart failure patients with lower HRQOL express greater interest in a smartphone app for heart failure than those with higher HRQOL. As the MLHFQ is reliable and sensitive to differences in symptom severity [29], heart failure patients with lower MLHFQ scores are likely more prominently afflicted by heart failure. Therefore, their interest in receiving heart failure—related information and reminders may suggest a greater likelihood of utilizing it as an individual-tailored intervention.

Analysis of age was a key aspect of this study because both prevalence and incidence of heart failure increase with age [6,7]. Accordingly, older heart failure patients are the primary target population for any intervention. In contrast to the increase of their interests with MLHFQ score, heart failure patients’ interest significantly decreased with age. This result is consistent with and can be explained by previous studies that examined adults’ technology usage and attitudes. In those studies, older adults acknowledged the benefits of technological advances but expressed several issues with technology, such as lack of security and reliability as well as inconvenience [30,31]. In addition, they identified low self-efficacy, high anxiety, and increased efforts as reasons for their reluctance to adopt technology [32,33]. As a result, their unfavorable outlook on technology poses a challenge to the prospect of implementing the smartphone app as an intervention. Providing incentives or alternatives, however, could address this challenge for those who may not be interested in mHealth apps.

Questionnaire scores from this survey revealed unexpected results. Both the MLHFQ and KCCQ were intended to quantify patients’ HRQOL with respect to their heart failure but revealed contrasting results with statistical significance (*P*<.001). The MLHFQ generated a median score that corresponded to poor HRQOL, whereas the KCCQ produced a median score that suggested good HRQOL. This discrepancy may be because of the fact that questions in the KCCQ examined a much shorter time frame (2 weeks) than those in the MLHFQ (4 weeks). Furthermore, the KCCQ is primarily concerned with 2 symptoms of heart failure, shortness of breath and fatigue, whereas the MLHFQ is more general. The scores from questionnaires regarding behavior and function produced mixed results, whereas all those regarding symptoms generated scores that fell within the normal range (Table 5). This outcome suggests that the mental health conditions of the participants were in favorable states despite their adverse health effects from heart failure. This finding appears to not align with a previous study that found heart failure patients have higher levels of anxiety than healthy adults, which leads to decreased treatment adherence [34]. The normal mental health of the participants may have influenced their interests in the smartphone app as a self-care strategy.

### Limitations and Future Directions

This study was confined to patients from a university-based health system and was limited to those aged between 50 and 80 years. The study population was relatively well educated, which might limit the generalization of our results, although we note that we did not observe any statistically significant correlations across the observed education levels with other variables. There was greater representation of male (34/50, 68%) and white (32/48, 67%) patients in the study cohort (Table 1), which might have generated results that are not applicable to the general population with heart failure. A reason for the disproportionate representation is that this study was limited to English language speakers. Literacy in English was necessary to understand the directions and questions because there was only an English version of the survey. Future study will include translation of this survey into other languages, particularly Spanish. In regard to the results, the statistically significant correlations do not indicate causation. Self-reporting of interest in mHealth may not translate to actual use, adherence, or persistence. Prospective testing of mobile technology apps will be needed along with evaluation of their effectiveness, safety, and value.

### Conclusions

This study provides new information on the features that heart failure patients want from a smartphone app to assist them in managing their health. To better contextualize the desired information and features, we sought to correlate survey responses, disease state, and demographics. On the basis of our results, we propose that a smartphone app may be a viable minimally invasive alternative intervention for monitoring heart failure patients because of the generally positive reception, although we note that data in this study were collected from a single site. Participants were interested in all 3 features of the proposed smartphone app—tracking, tips, and reminders. As these are common features of activity trackers and smartwatches, they, along with a smartphone app, may be potential solutions for heart failure patients’ self-care needs. Age and MLHFQ scores may be useful predictors in determining whether an heart failure patient is interested in a smartphone app for self-care. These findings suggest that certain populations may be more inclined to utilize mobile technology to manage their treatment and symptoms. We suggest that future mHealth-driven interventions that feature a smartphone app consider first soliciting feedback from their targeted population to better understand patient perspectives on how such technology can be designed to maximize impact. We suggest that this study is a step in this direction.

## Appendix

Multimedia Appendix 1 Questions in mobile health survey.

Multimedia Appendix 2 Ownership of mobile technology among participants interested in mobile health features.

## Abbreviations

EF: ejection fraction
HRQOL: health-related quality of life
IQR: interquartile range
KCCQ: Kansas City Cardiomyopathy Questionnaire
mHealth: mobile health
MLHFQ: Minnesota Living with Heart Failure Questionnaire
NYHA: New York Heart Association
PRO: patient-reported outcome
PROMIS: Patient-Reported Outcomes Measurement Information System
SAQ: Seattle Angina Questionnaire
SCHFI: Self-Care of Heart Failure Index
SF: short form
