# Supplementary material for: Assessment of Heart Failure Patients’ Interest in Mobile Health Apps for Self-Care: Survey Study
Source: JMIR Cardio. 2019 Oct 29;3(2):e14332. doi: 10.2196/14332 (PMC6851712; doi:10.2196/14332)
Supplement: Multimedia Appendix 1 [file cardio_v3i1e14332_app1.docx]

# Appendix

Supplemental Table 1: Questions in mHealth Survey

| Section Title, Question No. | Question |
| --- | --- |
| Demographics |  |
| 1 | Sex |
| 2 | Hispanic or Spanish Origin |
| 3 | Race or Ethnicity |
| 4 | Education |
| 5 | Annual Income |
| HF Self-Care Management Application Interest |  |
| 1 | Symptom identification, such as noticing swelling in your ankles or legs |
| 2 | Providing symptom management tips |
| 3 | Providing medication reminders |
| 4 | Documenting when you experience side effects from medication or treatment |
| 5 | Documenting your level of activity/number of steps |
| 6 | Providing reminders to get more exercise |
| 7 | Documenting your sleep patterns |
| 8 | Providing tips to get better sleep |
| 9 | Documenting your mood |
| 10 | Providing tips to improve your mood |
| HF Self-Care Management Application Engagement |  |
| 1 | Notify you of symptoms |
| 2 | Provide you with symptom management tips |
| 3 | Provide you with medication reminders |
| 4 | Provide you with your level of activity/number of steps |
| 5 | Provide you with exercise reminders |
| 6 | Provide you with sleep tips |
| HINTS |  |
| 1 | Do you ever go online to access the Internet or World Wide Web, or to send and receive e-mail? |
| 2 | When you use the Internet, do you ever access it through a regular dial-up telephone line? |
| 3 | When you use the Internet, do you ever access it through Broadband such as DSL, cable or FiOS? |
| 4 | When you use the Internet, do you ever access it through a cellular network (i.e., phone, 3G/4G)? |
| 5 | When you use the Internet, do you ever access it through a wireless network (Wi-Fi)? |
| 6 | Do you own a tablet? |
| 7 | Do you own a smartphone? |
| 8 | If so, do you use your smartphone at least once daily? |
| 9 | Do you own a cell phone? (skip if yes answer to smartphone) |
| 10 | If so, are you comfortable using the cell phone? |
| 11 | Do you own an activity tracker/smartwatch? |
| 12 | If so, do you wear it daily? |
