# Supplementary material for: Assessment of Heart Failure Patients’ Interest in Mobile Health Apps for Self-Care: Survey Study
Source: JMIR Cardio. 2019 Oct 29;3(2):e14332. doi: 10.2196/14332 (PMC6851712; doi:10.2196/14332)
Supplement: Multimedia Appendix 2 [file cardio_v3i1e14332_app2.docx]

Supplemental Table 2: Ownership of mobile technology among participants interested in mHealth features

| Feature | Interested^a^ | Smartphone^b^ | Activity Tracker or Smartwatch^b^ |
| --- | --- | --- | --- |
| Symptoms (*n* = 48) | 30 (62.5) | 28 (93.3) | 10 (33.3) |
| Medication or Treatment (*n* = 48) | 25 (52.1) | 23 (92.0) | 7 (28.0) |
| Activity (*n* = 48) | 28 (58.3) | 26 (92.9) | 11 (39.3) |
| Sleep (*n* = 46) | 27 (58.7) | 24 (88.9) | 11 (40.7) |
| Mood (*n* = 48) | 22 (45.8) | 20 (90.9) | 8 (36.4) |

Note—Data are number (%) of responses.

^a^ Interested denotes participants who answered either somewhat interested or very interested to both statements in HF Self-Care Management Application Interest corresponding to each feature.

^b^ Percentages are with respect to the number of interested participants.
